# Association of Inflammatory Gene Polymorphisms and Conventional Risk Factors With Arterial Stiffness by Age

**DOI:** 10.2188/jea.JE20130054

**Published:** 2013-11-05

**Authors:** Motahare Kheradmand, Hideshi Niimura, Kazuyo Kuwabara, Noriko Nakahata, Akihiko Nakamura, Shin Ogawa, Eva Mariane Mantjoro, Keiichi Shimatani, Yasuhito Nerome, Tetsuhiro Owaki, Ken Kusano, Toshiro Takezaki

**Affiliations:** 1 Department of International Islands and Community Medicine, Kagoshima University Graduate School of Medical and Dental Sciences, Kagoshima, Japan; 1 鹿児島大学大学院医歯学総合研究科国際島嶼医療学講座; 2 Education Center for Doctors in Remote Islands and Rural Areas, Kagoshima University Graduate School of Medical and Dental Sciences, Kagoshima, Japan; 2 鹿児島大学大学院医歯学総合研究科離島へき地医療人育成センター; 3 Kagoshima Prefectural Ohshima Hospital, Kagoshima, Japan; 3 鹿児島県立大島病院; 4 JA Kagoshima Kouseiren Medical Health Care Center, Kagoshima, Japan; 4 JA鹿児島県厚生連健康管理センター

**Keywords:** atherosclerosis, gene polymorphism, inflammation, cardio-ankle vascular index

## Abstract

**Background:**

Inflammatory gene polymorphisms are potentially associated with atherosclerosis risk, but their age-related effects are unclear. To investigate the age-related effects of inflammatory gene polymorphisms on arterial stiffness, we conducted cross-sectional and 5-year follow-up studies using the cardio-ankle vascular index (CAVI) as a surrogate marker of arterial stiffness.

**Methods:**

We recruited 1850 adults aged 34 to 69 years from the Japanese general population. Inflammatory gene polymorphisms were selected from *NF-kB1*, *CD14*, *IL-6*, *IL-10*, *MCP-1*, *ICAM-1*, and *TNF-α*. Associations of CAVI with genetic and conventional risk factors were estimated by sex and age group (34–49, 50–59, and 60–69 years) using a general linear model. The association with 5-year change in CAVI was examined longitudinally.

**Results:**

Glucose intolerance was associated with high CAVI among women in all age groups, while hypertension was associated with high CAVI among participants in all age groups, except younger women. Mean CAVI for the *CD14* CC genotype was lower than those for the TT and CT genotypes (*P* for trend = 0.005), while the *CD14* polymorphism was associated with CAVI only among men aged 34 to 49 years (*P* = 0.006). No association of the other 6 polymorphisms with CAVI was observed. No association with 5-year change in CAVI was apparent.

**Conclusions:**

Inflammatory gene polymorphisms were not associated with arterial stiffness. To confirm these results, further large-scale prospective studies are warranted.

## Abstract (ja)

**【背景】:**

炎症に関わる遺伝子多型は動脈硬化リスクに関与している可能性があるが、年齢別の効果については不明である。年齢別の動脈硬化（arterial stiffness）における遺伝子多型の効果を検索するために、cardio-ankle vascular index (CAVI)を動脈硬度の代理指標として用い、横断的および5年間の縦断的研究を行った。

**【方法】:**

対象は34～69歳の一般住民1,850名である。炎症に関わる遺伝子多型は*NF-kB1、CD14、IL-6、IL-10、MCP-1、ICAM-1、TNF-α*から選ばれた。CAVI値と遺伝および主要危険要因との関連は、一般線形モデルを用いて性・年齢群（34–49、50–59、60–69歳)ごとに見積もった。5年間におけるCAVI値の変化との関連も同様に縦断的に検討した。

**【結果】:**

耐糖能異常は女性の全年齢群で高CAVI値と関連していた。一方、高血圧も女性若年群以外の男女全年齢群でCAVI値と正の関連があった。遺伝子多型ごとのCAVI値は、男性ではCD14 C-260T多型のCC型で最も低い値を示し（*P* for trend = 0.005）、主要危険要因で調整した重回帰分析では、男性若年群におけるCD14 C-260T多型がCAVI値と正の関連を認めた（*P* = 0.006）。他の6遺伝子多型に関しては、CAVI値との関連は認められなかった。5年間のCAVI値の変化に関しては、明確な関連が認められなかった。

**【結論】:**

本研究では炎症に関わる遺伝子多型は動脈硬化と関連していないことが示唆された。この結果を確認するためには、さらに大規模な追跡研究が必要である。

## INTRODUCTION

Atherosclerosis develops through a complex multifactorial process involving multiple pathways that are influenced by genetic and environmental factors.^1^ Inflammation is involved in this process from inception to the final complication of thrombosis,^2^^,^^3^ as are conventional risk factors such as hypertension, dyslipidemia, diabetes mellitus, and smoking.^1^^,^^4^^,^^5^

Various cellular and molecular responses are involved in atherogenesis-related inflammation.^6^ CD14 is a pattern-recognition molecule involved in the innate immune response and acts as the main receptor for lipopolysaccharides, endogenous heat shock proteins (HSP60), and oxidized low-density lipoprotein cholesterol (LDL-C), which elicit inflammatory responses that function with toll-like receptors (TLRs).^7^^,^^8^ CD14 acts as a trigger in the production of cytokines and other molecules.^7^ A functional polymorphism in the promoter region of *CD14* at position -260 (the *CD14* C-260T polymorphism) enhances the transcriptional activity of the *CD14* gene and was found to be associated with increased carotid artery intima-media thickness (IMT) and elevated risk of stroke and acute myocardial infarction.^8^^,^^9^ A recent meta-analysis revealed that the *CD14* C-260T polymorphism is a risk factor for coronary heart disease, especially in East Asians.^10^

Binding of ligands to the TLR4/CD14 complex initiates activation of nuclear factor-κB (NF-κB) and induces secretion of proinflammatory cytokines such as interleukin (IL)-1β, IL-6,^11^ tumor necrosis factor-α (TNF-α), and growth factors.^12^ The P50 subunit of NF-κB, which is encoded by *NF-κB1*, represses transcription of proinflammatory cytokines and stimulates transcription of the anti-inflammatory cytokine IL-10.^13^^,^^14^ In contrast, macrophages proliferate and amplify the inflammatory response by secreting numerous growth factors and cytokines that induce intercellular adhesion molecule-1 (ICAM-1) and monocyte chemoattractant protein-1 (MCP-1).^15^ ICAM-1 and MCP-1 also initiate the inflammatory process of atherogenesis and sustain the proliferative response within the vessel wall.^16^^–^^20^ Several inflammatory gene polymorphisms were found to be associated with increased risk of atherosclerosis and its related diseases, but such reports were mainly limited to analysis of *IL-6*, *IL10*, *ICAM-1*, *TNF-α*, *MCP-1*, and *NF-κB1*.^2^^,^^17^^–^^22^

Age is a risk factor for atherosclerosis development. Several studies have reported that patients who develop cardiovascular diseases and stroke at a relatively young age have a higher potential for genetic factors.^23^^,^^24^ Although the prevalence of conventional risk factors generally increases with age, the atherogenesis-related effects of genetic alterations may differ by age. To our knowledge, only a few studies have investigated the effect of genetic alterations specific to atherosclerosis development by age. The cardio-ankle vascular index (CAVI) is a recently developed method that measures pulse-wave velocity (PWV) to determine the stiffness of the aorta and femoral and tibial arteries. Its advantages include the fact that it is less influenced by blood pressure as compared with previous methods of PWV measurement.^25^^–^^27^ CAVI allows researchers to assess arterial stiffness at a relatively early phase of atherosclerosis, ie, before development of coronary heart disease (CHD). To investigate the effect of inflammatory gene polymorphisms on arterial stiffness by age, we conducted cross-sectional and 5-year follow-up studies using CAVI as a surrogate marker of arterial stiffness.

## METHODS

### Population

The study participants were recruited from a portion of the population-based cohort study (Japan Multi-Institutional Collaborative Cohort Study [J-MICC study]), which has been described elsewhere.^28^^–^^30^ The baseline survey was conducted in 3 island regions of Amami, Kagoshima prefecture, Japan, in 2005 and 2006. We recruited 3194 Japanese adults aged 34 to 69 years at baseline who underwent a routine health checkup that was conducted by the local government or a private company after written informed consent was obtained.

The baseline survey consisted of a questionnaire survey, blood collection, and examination of arterial stiffness using CAVI. The same participants were asked to take part in a second survey, 5 years later, in 2010 and 2011 at another routine health checkup, and 2076 agreed to participate (65.0% of initial participants). Most participants who did not take part in the second survey had not undergone a routine health checkup, either because of changes in the health checkup system or for personal reasons. The present participants were selected from the men and women (for both the baseline and second surveys) among whom data from the questionnaire, blood samples, health checkup, and CAVI were obtained with informed consent. We excluded individuals with dysrhythmias such as bradycardia, atrial fibrillation, and frequent arterial or ventricular premature contractions; a history of surgery for arteriosclerosis obliterans; Parkinson disease; or an ankle brachial pressure index of less than 0.9, because these conditions cause errors in CAVI measurement. Ultimately, 1850 participants were included in the analysis.

The present study was approved by the ethics review committee for human genome/gene analysis research of the Kagoshima University Graduate School of Medical and Dental Sciences, Kagoshima, Japan.

### Questionnaire

We used a structured questionnaire that was standardized by the J-MICC study.^28^ The questionnaire collected information on smoking, drinking, exercise, dietary habits, medical history, intake of supplements and prescription medicines, reproductive history, and stress status.

### Health checkup

We used heath checkup data from the Kagoshima Kouseiren Health Center. These data included systolic and diastolic blood pressure and levels of total cholesterol, triglyceride (TG), high-density lipoprotein cholesterol (HDL-C), fasting blood sugar (FBS), blood urea nitrogen, creatinine, and uric acid. Participants were asked to fast for at least 10 hours. LDL-C levels were calculated according to the Friedewald formula, using a TG level of less than 400 mg/dL.^31^

### CAVI measurement

We used the VaSera VS-1000 and VS-1500 vascular screening systems (Fukuda Denshi Co., Ltd., Tokyo, Japan) to measure CAVI, as described elsewhere.^25^^–^^27^ Briefly, cuffs were applied to both of the upper arms and ankles, electrocardiogram leads were attached to both wrists, and a phonocardiogram was placed on the sternum border in the second intercostal space. Participants were instructed to lay supine and hold their heads at the midline position. CAVI values were calculated using the formula CAVI = a[(2ρ/ΔP) × Ln(ps/pd) × PWV^2^] + b, where ρ is blood density, Ps is systolic blood pressure, Pd is diastolic blood pressure, ΔP is Ps − Pd, and a and b are constants that match aortic PWV. This equation was derived from the Bramwell–Hill equation and the stiffness parameter β.^25^

### DNA extraction

DNA was extracted from the buffy coat fraction using standard methods and a QIAamp Blood Mini-Kit (Qiagen, Valencia, CA, USA), GenElute Blood Genomic DNA Kit (Sigma-Aldrich, St. Louis, MO, USA), or Blood–Animal–Plant DNA Preparation Kit (Jena Bioscience, Jena, Germany).

### Genotyping single-nucleotide polymorphisms

We selected candidate single-nucleotide polymorphisms (SNPs) in genes related to the inflammatory mechanism of atherosclerosis on the basis of earlier studies that used minor allele frequencies of greater than 0.05, including *CD14* C-260T (rs2569190), *IL-10* T-819C (rs1800871), *IL-6* C-636G (rs1800796), *TNF-α* C-1031T (rs1799964), *ICAM-1* K469E (rs5498), and *MCP-1* A-2518G (rs1024611). We also selected the common insertion/deletion polymorphism ATTG (rs28362491), which is located in the *NF-κB1* gene. The genomic DNA samples were genotyped for these 7 SNPs using TaqMan allelic discrimination assays (Applied Biosystems, Foster City, CA, USA) in a real-time polymerase chain reaction (PCR) device (StepOne; Applied Biosystems). Each PCR reaction was performed in a 10-µL volume containing 1 µL of genomic DNA (10 ng/µL), 5 µL of TaqMan Universal PCR Master Mix II, 0.45 µL of 40× genotyping Assay Mix, and 3.75 µL of dH_2_O. The cycling was initiated by heating at 95°C for 10 minutes, followed by 40 cycles at 95°C for 15 seconds, 60°C for 1 minute, and 25°C for 30 seconds.

### Statistical analysis

We used 2 variables—systolic blood pressure (continuous) and antihypertensive medication—for hypertension because CAVI is strongly associated with systolic blood pressure^30^ and blood pressure is modified by medication at the time of examination. Dyslipidemia was defined as an LDL-C level of 140 mg/dL or higher, an HDL-C level of less than 40 mg/dL, a TG level of 150 mm Hg or higher, or use of antihyperlipidemic medication. Glucose intolerance was defined as an FBS value of 110 mg or higher or history of treatment for diabetes mellitus. Cigarette smoking was quantified on the basis of pack-years and categorized into 3 groups: never smoked, less than 20 pack-years, and 20 or more pack-years. Ex-smokers were included among never smokers. Dietary intakes of fish, fruit, chicken, pork, green vegetables, green tea, and coffee were categorized into 3 groups according to intake frequency, with almost equal numbers of participants in each group.

Hypertension, dyslipidemia, glucose intolerance, family history of CHD and stroke, and smoking habit were analyzed as conventional risk factors. The conventional risk factors and lifestyles of men and women were compared with respect to median CAVI using the 2-sided *t* test and χ^2^ test. A trend test was performed to compare mean CAVI by genotype to observe the effect of the SNPs in an additive or a threshold model. A general linear model was used to assess the associations between conventional and genetic factors, as well as CAVI, after adjustment for age, region, body mass index (BMI), and conventional factors. A score of 1 to 3 (reflecting the number of high-risk alleles, as determined by the findings of previous studies) was used for all SNP analyses.^2^^,^^12^^,^^21^^–^^26^

To observe differences in genetic and environmental factors in relation to CAVI by age, we conducted a subgroup analysis and classified participants into 3 age groups: 34 to 49, 50 to 59, and 60 to 69 years. Regions were included for the adjustment variable because the frequencies of the genotypes differed by region. *P* values less than 0.05 were considered statistically significant. We applied Bonferroni correction in the SNP analysis to decrease the potential of an α error in multiple hypothesis testing. *P* values less than 0.007 were considered nominally significant in the analysis of the 7 SNPs. The statistical calculations were performed using Stata software version 10 (Stata Corp., College Station, TX, USA). Genotypes with distributions that differed from the Hardy-Weinberg equilibrium (HWE) were assessed using the Pearson χ^2^ test with the “genhwi” command in Stata.

## RESULTS

Table 1 shows the baseline characteristics of the participants, according to sex and median CAVI. Median CAVI was 8.03 among men and 7.66 among women. As compared with men and women with a CAVI below the median, those with a median or higher CAVI were older, had higher systolic and diastolic blood pressures, were more likely to be taking antihypertensive medication, be glucose intolerant, and engage in habitual exercise, and had a higher green tea intake. Similarly, a family history of CHD or stroke and current drinking were more common, and fish intake was higher, among men with a median or higher CAVI, and dyslipidemia was more frequent among women with a median or higher CAVI. To assess the possibility of selection bias due to exclusion of participants who did not take part in the second survey, we compared the baseline characteristics of nonparticipants and participants. The differences were not significant (data not shown).

**Table 1.** Baseline characteristics of study participants by sex

	Men (*n* = 744)			Women (*n* = 1106)		
		
	CAVI < median	CAVI ≥ median	*P* value	CAVI < median	CAVI ≥ median	*P* value
Age (years)^a^	51.4 (7.7)	58.6 (7.8)	<0.001	51.9 (7.5)	58.4 (6.7)	<0.001
CAVI at baseline^a^	7.30 (0.52)	8.86 (0.61)	<0.001	6.98 (0.55)	8.47 (0.63)	<0.001
CAVI at 5-year follow-up^a^	7.94 (0.88)	9.23 (0.91)	<0.001	7.70 (0.77)	8.91 (0.92)	<0.001
Systolic blood pressure^a^	130.4 (15.8)	138.5 (17.8)	<0.001	121.0 (15.8)	131.2 (18.5)	<0.001
Diastolic blood pressure^a^	81.9 (10.7)	85.2 (11.0)	<0.001	74.4 (10.7)	79.4 (10.8)	<0.001
Use of antihypertensive medication^b^	51 (13.6)	127 (34.3)	<0.001	69 (12.4)	165 (30.0)	<0.001
Dyslipidemia^b^	197 (52.7)	208 (56.2)	0.332	234 (42.2)	312 (56.6)	<0.001
Glucose intolerance^b^	64 (17.1)	111 (30.0)	<0.001	34 (6.1)	85 (15.5)	<0.001
Family history of CHD or stroke^b^	97 (28.5)	111 (36.4)	0.031	151 (30.4)	171 (36.0)	0.063
Smoking (≥20 pack-years)^b^	95 (25.5)	92 (24.9)	0.700	8 (1.4)	6 (1.1)	0.554
Drinking (≥3 times/week)^b^	250 (66.84)	281 (75.95)	<0.001	88 (15.86)	73 (13.25)	0.130
Fish intake (≥3 times/week)^b^	162 (43.4)	188 (51.0)	0.010	278 (50.5)	312 (57.4)	0.167
Green vegetable intake (≥3 times/week)^b^	128 (34.5)	124 (33.7)	0.753	246 (44.7)	274 (50.4)	0.062
Green tea intake (≥3 times/week)^b^	74 (20.0)	122 (33.5)	<0.001	187 (34.1)	253 (46.3)	<0.001
Habitual exercise (≥3 times/week)^b^	116 (31.0)	156 (42.2)	<0.001	200 (36.0)	237 (43.2)	0.019

Median CAVI was 8.03 among men and 7.66 among women.

Family history included 232 missing values.

CAVI, cardio-ankle vascular index; CHD, coronary heart disease.

^a^Data are presented as mean (SD).

^b^Data are presented as number (%).

The minor allele frequencies (MAFs) for the selected 7 SNPs are shown in Table 2. MAF ranged from 19%, for the *TNF-α* C-1031T polymorphism, to 47%, for the *CD14* C-260T polymorphism. Excepting *MCP-1* (A-2518G, *P* = 0.001) and *CD14* (C-260T, *P* = 0.009), the genotypes were in HWE. Twenty genotypes were independently undetectable (4 in *IL-10*, 5 in *ICAM-1*, 5 in *TNF-α*, 3 in *CD14*, and 3 in *NF-κB1*) among the 1850 participants. The genotype frequencies of *MCP-1* and *CD14* were also compared by region. Their frequencies were out of the HWE in 1 region, while the other 2 regions had frequencies within the HWE.

**Table 2.** Single-nucleotide polymorphism characteristics among study participants

Gene	rs number	Alias	Allele	MAF	HWE *P* value
*IL-6*	rs1800796	C-634G	C > G	0.35	0.068
*IL-10*	rs1800871	T-819C	T > C	0.43	0.287
*ICAM-1*	rs5498	K469E	A > G	0.37	0.691
*TNF-α*	rs1799964	C-1031T	T > C	0.19	0.337
*MCP-1*	rs1024611	A-2518G	G > A	0.36	0.001
*CD14*	rs2569190	C-260T	T > C	0.47	0.009
*NF-kB1*	rs28362491	ins/del ATTG	ins > del	0.35	0.082

MAF, minor allele frequency; HWE, Hardy-Weinberg equilibrium; IL-6, interleukin-6; IL-10, interleukin-10; ICAM-1, intracellular adhesion molecule-1; TNF-α, tumor necrosis factor-α; MCP-1, monocyte chemoattractant protein-1; NF-κB1, nuclear factor-κ B1.

**Figure 1.**
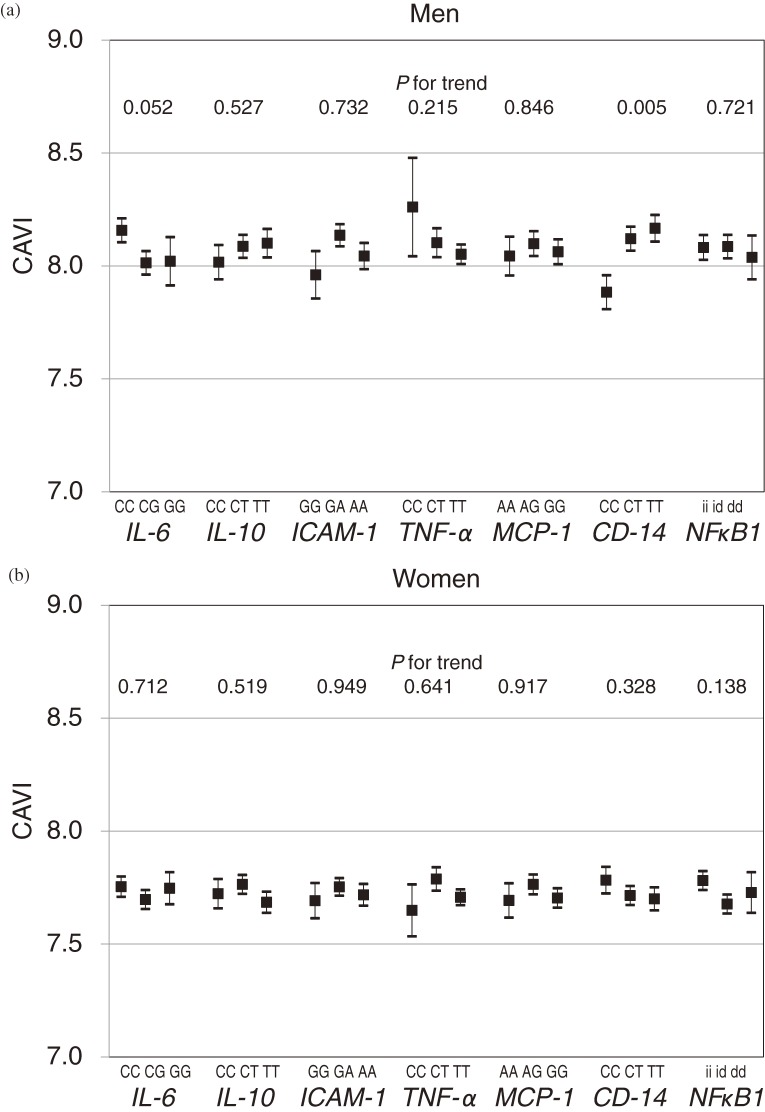
(a) Mean cardio-ankle vascular index (CAVI) values by single-nucleotide polymorphism genotype among men. (b) Mean cardio-ankle vascular index (CAVI) values by single-nucleotide polymorphism genotype among women. ■, mean CAVI; lines indicate standard error. *P* values were tested using the trend test. IL-6, interleukin-6; IL-10, interleukin-10; TNF-α, tumor necrosis factor-α; MCP-1, monocyte chemoattractant protein-1; NFκB1, nuclear factor-κ B1.

Mean CAVI did not differ by genotype among the 6 SNPs, among men or women, except for *CD14* C-260T in men (Figures 1a, 1b). Among men, the mean CAVI for the *CD14* CC genotype was lower than those for the TT and CT genotypes in the threshold model (*P* for trend = 0.005) and was the lowest of any genotype for the 7 SNPs. CAVI values among women were always lower than those among men for the same genotype and SNP (Figures 1a, 1b).

Associations of genetic and conventional risk factors with CAVI were analyzed by sex and age group, using the general linear model. CAVI was positively associated with systolic blood pressure among men and women in the older age groups (50–59 and 60–69 years) and was associated with use of antihypertensive medication among men in the youngest age group (34–49 years; Table 3). Among both men and women, dyslipidemia was related to higher CAVI when all age groups were combined. Glucose intolerance was positively associated with CAVI among women in all age groups and among men aged 50 to 59 years. An inverse association between smoking and CAVI was observed among women aged 60 to 69 years, but there were few smokers in this subgroup (*n* = 14). A significant association between a SNP and higher CAVI was observed only for the *CD14* C-260T polymorphism (CC, CT, and TT genotypes in order of scoring) among men aged 34 to 49 years (coefficient = 0.190, *P* = 0.006) after Bonferroni correction. We compared mean systolic and diastolic blood pressures and the prevalences of other conventional factors by CD14 genotype among men and women in each age group; no significant difference was observed (data not shown).

**Table 3.** Correlation coefficients between CAVI and conventional and genetic factors by sex and age

	Men								Women							
		
	34–49 yrs		50–59 yrs		60–69 yrs		Total		34–49 yrs		50–59 yrs		60–69 yrs		Total	
								
	*n* = 219		*n* = 265		*n* = 260		*n* = 744		*n* = 298		*n* = 464		*n* = 344		*n* = 1106	
	Coef.	*P*	Coef.	*P*	Coef.	*P*	Coef.	*P*	Coef.	*P*	Coef.	*P*	Coef.	*P*	Coef.	*P*
Conventional factors																
Systolic blood pressure, mm Hg	0.002	0.409	0.007	0.020	0.010	0.001	0.000	<0.001	0.003	0.310	0.006	0.010	0.010	<0.001	0.000	<0.001
Use of antihypertensive medication, no/yes	0.710	<0.001	0.230	0.090	0.170	0.188	0.300	<0.001	0.150	0.318	0.190	0.080	0.162	0.202	0.170	0.014
Dyslipidemia, no/yes	0.150	0.171	0.080	0.410	0.200	0.083	0.150	0.020	0.136	0.143	0.093	0.026	0.173	0.127	0.130	0.014
Glucose intolerance, no/yes	0.100	0.510	0.450	0.001	0.201	0.117	0.270	<0.001	0.400	0.020	0.360	0.004	0.378	0.034	0.399	<0.001
Family history of CHD or stroke, no/yes	0.010	0.890	0.060	0.555	0.201	0.117	0.050	0.406	0.112	0.200	0.030	0.658	−0.095	0.394	0.000	0.980
Smoking status, never/<20PY/≥20PY	0.090	0.150	0.080	0.222	0.010	0.861	0.050	0.138	−0.070	0.480	0.090	0.490	−0.560	0.010	−0.090	0.265
Genetic factors																
*IL-6* C-634G (CC, CG, GG)	−0.040	0.542	−0.181	0.023	0.060	0.434	−0.050	0.200	0.080	0.137	−0.040	0.473	0.010	0.860	0.010	0.652
*IL-10* T-819C (CC, CT, TT)	0.060	0.437	0.107	0.150	0.029	0.718	0.060	0.126	0.010	0.820	−0.040	0.453	−0.070	0.325	−0.030	0.329
*ICAM-1* K469E (GG, GA, AA)	0.040	0.527	−0.090	0.220	0.050	0.524	0.000	0.833	0.002	0.965	0.006	0.904	0.040	0.563	0.010	0.705
*TNF-α* C-1031 (CC, CT, TT)	0.110	0.251	−0.080	0.380	0.020	0.810	0.000	0.994	0.020	0.680	−0.080	0.237	0.110	0.251	0.000	0.903
*MCP-1* A-2518G (AA, AG, GG)	0.030	0.678	0.020	0.770	−0.125	0.124	−0.010	0.739	−0.040	0.430	0.040	0.426	−0.050	0.449	0.000	0.826
*CD14* C-260T (CC, CT, TT)	0.190	0.006	−0.003	0.950	0.030	0.644	0.068	0.110	−0.030	0.502	0.000	0.946	−0.090	0.231	−0.020	0.413
*NF-κB1* ATTG (ins/ins, ins/del, del/del)	0.020	0.766	0.060	0.371	−0.090	0.279	0.000	0.989	0.030	0.518	−0.090	0.116	−0.090	0.252	−0.060	0.100

Coefficients in the general linear model were adjusted for age, region, body mass index, systolic blood pressure, use of antihypertensive medication, dyslipidemia, glucose intolerance, family history, and smoking status.

CAVI, cardio-ankle vascular index; Coef., coefficient; PY, pack-years; IL-6, interleukin-6; IL-10, interleukin-10; ICAM-1, intracellular adhesion molecule-1; TNF-α, tumor necrosis factor-α.

We longitudinally estimated the effect of conventional and genetic factors on 5-year change in CAVI between the baseline and second surveys. Among men aged 34 to 49 years, systolic blood pressure was positively associated with CAVI change, but use of antihypertensive medication was negatively associated with CAVI change (Table 4). An inverse association was observed between dyslipidemia and CAVI change among women aged 50 to 59 years. Glucose intolerance was associated with increased CAVI change among men aged 34 to 59 years. Smoking was associated with increased CAVI change among women aged 60 to 69 years. No significant association with the 7 gene polymorphisms was observed after Bonferroni correction.

**Table 4.** Correlation coefficients between 5-year change in CAVI and conventional and genetic factors by sex and age

	Men								Women							
		
	34–49 yrs		50–59 yrs		60–69 yrs		Total		34–49 yrs		50–59 yrs		60–69 yrs		Total	
								
	*n* = 219		*n* = 265		*n* = 260		*n* = 744		*n* = 298		*n* = 464		*n* = 344		*n* = 1106	
	Coef.	*P*	Coef.	*P*	Coef.	*P*	Coef.	*P*	Coef.	*P*	Coef.	*P*	Coef.	*P*	Coef.	*P*
Conventional factors																
Systolic blood pressure, mm Hg	0.008	0.020	0.002	0.394	−0.006	0.073	0.001	0.528	0.005	0.096	0.009	<0.001	0.005	0.108	0.003	0.023
Use of antihypertensive medication, no/yes	−0.630	0.001	0.167	0.234	−0.023	0.867	−0.080	0.338	0.207	0.214	0.048	0.657	−0.046	0.709	0.011	0.874
Dyslipidemia, no/yes	−0.010	0.917	−0.120	0.275	0.097	0.420	−0.074	0.267	0.114	0.243	−0.232	0.006	0.244	0.029	0.148	0.007
Glucose intolerance, no/yes	0.398	0.012	−0.249	0.064	0.122	0.345	0.033	0.673	−0.187	0.322	−0.040	0.748	0.089	0.609	0.035	0.694
Family history of CHD or stroke, no/yes	0.077	0.526	0.098	0.391	0.010	0.929	0.033	0.673	−0.114	0.243	−0.074	0.390	0.011	0.920	0.049	0.378
Smoking status, never/<20PY/≥20PY	0.127	0.059	0.026	0.687	0.091	0.258	0.012	0.757	0.188	0.102	−0.003	0.980	0.547	0.012	0.160	0.062
Genetic factors																
*IL-6* C-634G (CC, CG, GG)	0.130	0.082	0.093	0.246	−0.037	0.654	0.010	0.813	−0.020	0.680	0.032	0.585	0.020	0.806	−0.007	0.843
*IL-10* T-819C (CC, CT, TT)	0.033	0.671	0.032	0.668	0.070	0.392	0.049	0.285	0.058	0.320	0.053	0.339	0.012	0.866	0.047	0.199
*ICAM-1* K469E (GG, GA, AA)	0.001	0.988	−0.125	0.119	−0.033	0.704	0.046	0.331	0.115	0.052	−0.017	0.761	0.079	0.300	0.051	0.169
*TNF-α* C-1031 (CC, CT, TT)	0.025	0.803	0.024	0.812	−0.089	0.371	0.000	0.990	−0.111	0.111	0.035	0.606	0.062	0.515	−0.003	0.941
*MCP-1* A-2518G (AA, AG, GG)	0.047	0.532	0.050	0.499	0.114	0.168	0.058	0.193	0.169	0.006	−0.047	0.422	0.001	0.983	0.026	0.478
*CD14* C-260T (CC, CT, TT)	−0.022	0.755	0.140	0.063	0.069	0.395	0.024	0.581	0.006	0.907	0.026	0.624	0.072	0.324	0.027	0.446
*NF-κB1* ATTG (ins/ins, ins/del, del/del)	0.022	0.772	−0.064	0.395	0.046	0.585	0.004	0.921	0.032	0.593	0.018	0.759	0.071	0.353	0.032	0.395

Coefficients in the general linear model were adjusted for age, region, body mass index, systolic blood pressure, use of antihypertensive medication, dyslipidemia, glucose intolerance, family history, and smoking status.

CAVI, cardio-ankle vascular index; Coef., coefficient; PY, pack-years; IL-6, interleukin-6; IL-10, interleukin-10; ICAM-1, intracellular adhesion molecule-1; TNF-α, tumor necrosis factor-α.

## DISCUSSION

We investigated the association of CAVI with conventional risk factors and inflammatory genetic factors by age group in a cross-sectional study and a 5-year prospective study. We found positive associations between high CAVI and glucose intolerance among women in all age groups. In addition, hypertension was associated with CAVI in all age groups except younger women. Associations between inflammatory gene polymorphisms and CAVI were not obvious, except for an association with the *CD14* polymorphism among men aged 34 to 49 years. This is the first study to observe an association between an inflammatory gene polymorphism and arterial stiffness in analysis stratified by age.

Epidemiologic studies have proven that age is the dominant risk factor for cardiovascular disease.^32^ Several studies have shown a linear correlation between IMT and age.^33^^,^^34^ The age-related decrease in arterial compliance is due both to deterioration in the integrity of elastic load-bearing fibers and to atherosclerosis.^35^ The age-related effects of hypertension and hypercholesterolemia on IMT were reported to differ.^34^ Hypertension was a consistent and significant factor that affected almost all age groups and involved both initial and advanced lesions; however, hypercholesterolemia was a significant factor only among adults older than 50 years.

Development of preatheroma suggests a shift to the advanced stage of atherosclerosis. Hypertension possibly reduces the time required to reach this critical point regardless of age, while hypercholesterolemia accelerates atherosclerosis development after that point.^34^ Hypertensive individuals exhibit endothelial dysfunction, the underlying mechanisms of which are similar to those associated with aging among normotensive individuals, although they appear at an earlier age.^36^ The present study concordantly showed that hypertension was associated with increased CAVI in all age groups except younger women. CAVI was previously shown to correlate with IMT and was validated as a surrogate marker of arterial stiffness.^25^^–^^27^

High glucose levels were found to damage or alter the endothelial barrier, thus allowing insulin to interact with underlying smooth muscle cells.^37^ The impact of the association between glucose intolerance and early atherosclerosis was more evident among women than among men,^38^^,^^39^ although the reason for this disparity was not clear. The present study found a positive association between glucose intolerance and CAVI among men and women when all age groups were combined, but the association by age group was more obvious among women.

We observed no association between inflammatory gene polymorphisms and CAVI, except for an association with the *CD14* polymorphism among men aged 34 to 49 years. Studies in mice and humans showed that inflammation drives all phases of atherosclerosis, including initiation, progression, and thrombotic lesion complications.^3^ Various cellular and molecular responses are involved in atherogenesis-related inflammation and are related to each other in a complex manner.^6^ Furthermore, an individual polymorphism contributes only a small fraction to the entire heritable variance in protein concentration, even if the polymorphism is associated with protein function or concentration and protein concentration is associated with disease.^40^ The small contribution of a SNP to an individual’s overall risk of a multifactorial disorder such as atherosclerosis might be obscured by the presence of conventional risk factors. The present results suggest that the association of inflammatory gene polymorphisms with arterial stiffness may be too small to detect. Furthermore, relatively wide variation in CAVI values may decrease the ability to detect a small effect, because CAVI is a surrogate marker for atherosclerosis and the participants were recruited from a general population.

The impact of genetic factors on cardiovascular diseases and stroke also differs by age.^23^^,^^24^ A monozygotic twin study showed that early death from CHD was influenced by genetic factors and that the effects decreased with age.^41^ *CD14* was the first gene to be implicated in susceptibility to atherosclerotic disease.^42^ Endogenous HSP60 and oxidized LDL-C bind TLR4/CD14 complexes and elicit inflammatory responses.^8^ CD14 signaling has a fundamental role in mediating activation of mononuclear cells and macrophages in response to human and chlamydial HSP60.^43^

Activation of the TLR4/CD14 complex causes intracellular signaling cascades that trigger other proinflammatory cytokines. Therefore, the *CD14* polymorphism is a good candidate for assessing the genetic effect of atherosclerosis development at a relatively early phase. We observed a positive association between the *CD14* C-260T polymorphism and CAVI only among men aged 34 to 49 years. CAVI was lower among men with the CC genotype of the *CD14* C-260T polymorphism than among those with other genotypes and SNPs. However, these findings were not observed among women or other age groups of men, and the distribution of CD14 genotypes was outside the HWE. These findings suggest that our findings on the CD14 polymorphism were potentially due to chance. To confirm these results, further prospective studies with more participants are warranted.

We longitudinally investigated the effect of conventional risk factors and inflammatory gene polymorphisms on 5-year change in CAVI. The results did not accord with those of the present cross-sectional study or those of previous reports. The range of values for 5-year change in CAVI may be smaller than those obtained after the long-term accumulation observed in the cross-sectional study.

Several limitations of this study must be considered. First, we did not use CHD as the outcome; rather, we used CAVI as a surrogate marker of arterial stiffness. The use of CAVI is advantageous because it can assess arterial stiffness at a relatively early phase of atherosclerosis, before development of CHD. CAVI has been validated as a surrogate marker of arterial stiffness,^25^^–^^27^ although it might not be fully representative of atherosclerosis. Second, we selected 7 SNPs by referring to earlier reports on the inflammatory mechanisms of atherosclerosis. Numerous inflammatory genes may be involved in atherosclerosis development, but reports on their polymorphisms are limited mainly to the present SNPs. Third, the genotype frequencies of the *MCP-1* and *CD14* polymorphisms were not within the HWE, most likely due to genotyping error or population stratification. Because we tested the HWE in these SNPs by region and observed deviation in a different region among 3 regions, the chances of genotyping error are low. On the other hand, the study participants were selected from a general population with a common Japanese ethnicity, and information is lacking on the migration of the large-sized population in the study regions. We included the regions in the general linear model to adjust for this deviation.

In conclusion, inflammatory gene polymorphisms were not associated with arterial stiffness as represented by CAVI. The association was also unclear in age-stratified analysis. To confirm these results, further large-scale prospective studies are warranted.

## ONLINE ONLY MATERIALS

Abstract in Japanese.
