# Supplementary material for: Association of Inflammatory Gene Polymorphisms and Conventional Risk Factors With Arterial Stiffness by Age
Source: J Epidemiol. 2013 Nov 5;23(6):457–65. doi: 10.2188/jea.JE20130054 (PMC3834284; doi:10.2188/jea.JE20130054)
Supplement: Abstract in Japanese. [file je-23-457-s001.pdf]

炎症に関わる遺伝子多型および主要危険要因と年齢別の動脈硬化との関連に関する研究

Motahare Kheradmand[1]、新村英士[1]、桑原和代[2]、中畑典子[1]、中村昭彦[1]、小川信[3]、Eva Mariane Mantjoro[1]、嶋谷圭一[1]、根路銘安仁[2]、大脇哲洋[2]、草野健[4]、嶽崎俊郎[1]

[1]鹿児島大学大学院医歯学総合研究科国際島嶼医療学講座
[2]鹿児島大学大学院医歯学総合研究科離島へき地医療人育成センター
[3]鹿児島県立大島病院
[4]JA 鹿児島県厚生連健康管理センター

【背景】炎症に関わる遺伝子多型は動脈硬化リスクに関与している可能性があるが、年齢別の効果については不明である。年齢別の動脈硬化（arterial stiffness）における遺伝子多型の効果を検索するために、cardio-ankle vascular index (CAVI) を動脈硬度の代理指標として用い、横断的および 5 年間の縦断的研究を行った。
【方法】対象は 34～69 歳の一般住民 1,850 名である。炎症に関わる遺伝子多型は *NF-kB1*、*CD14*、*IL-6*、*IL-10*、*MCP-1*、*ICAM-1*、*TNF-α* から選ばれた。CAVI 値と遺伝および主要危険要因との関連は、一般線形モデルを用いて性・年齢群（34–49、 50–59、60–69 歳)ごとに見積もった。5 年間における CAVI 値の変化との関連も同様に縦断的に検討した。
【結果】耐糖能異常は女性の全年齢群で高 CAVI 値と関連していた。一方、高血圧も女性若年群以外の男女全年齢群で CAVI 値と正の関連があった。遺伝子多型ごとの CAVI 値は、男性では CD14 C-260T 多型の CC 型で最も低い値を示し（*P* for trend = 0.005）、主要危険要因で調整した重回帰分析では、男性若年群における CD14 C-260T 多型が CAVI 値と正の関連を認めた（*P* = 0.006）。他の 6 遺伝子多型に関しては、CAVI 値との関連は認められなかった。5 年間の CAVI 値の変化に関しては、明確な関連が認められなかった。
【結論】本研究では炎症に関わる遺伝子多型は動脈硬化と関連していないことが示唆された。この結果を確認するためには、さらに大規模な追跡研究が必要である。
